# Vaccinia Virus Uses Retromer-Independent Cellular Retrograde Transport Pathways To Facilitate the Wrapping of Intracellular Mature Virions during Virus Morphogenesis

**DOI:** 10.1128/JVI.01464-16

**Published:** 2016-10-28

**Authors:** Kate Harrison, Ismar R. Haga, Tali Pechenick Jowers, Seema Jasim, Jean-Christophe Cintrat, Daniel Gillet, Thomas Schmitt-John, Paul Digard, Philippa M. Beard

**Affiliations:** a The Roslin Institute and Royal (Dick) School of Veterinary Studies, University of Edinburgh, Roslin, Midlothian, United Kingdom; b The Pirbright Institute, Pirbright, Surrey, United Kingdom; c SCBM, Institute of Biology and Technology of Saclay, CEA, LabEx LERMIT, Université Paris-Saclay, Gif Sur Yvette, France; d SIMOPRO, Institute of Biology and Technology of Saclay, CEA, LabEx LERMIT, Université Paris-Saclay, Gif Sur Yvette, France; e Molecular Biology and Genetics, Aarhus University, Aarhus, Denmark; University of Florida

## Abstract

Poxviruses, such as vaccinia virus (VACV), undertake a complex cytoplasmic replication cycle which involves morphogenesis through four distinct virion forms and includes a crucial wrapping step whereby intracellular mature virions (IMVs) are wrapped in two additional membranes to form intracellular enveloped virions (IEVs). To determine if cellular retrograde transport pathways are required for this wrapping step, we examined VACV morphogenesis in cells with reduced expression of the tetrameric tethering factor known as the GARP (Golgi-associated retrograde pathway), a central component of retrograde transport. VACV multistep replication was significantly impaired in cells transfected with small interfering RNA targeting the GARP complex and in cells with a mutated GARP complex. Detailed analysis revealed that depletion of the GARP complex resulted in a reduction in the number of IEVs, thereby linking retrograde transport with the wrapping of IMVs. In addition, foci of viral wrapping membrane proteins without an associated internal core accumulated in cells with a mutated GARP complex, suggesting that impaired retrograde transport uncouples nascent IMVs from the IEV membranes at the site of wrapping. Finally, small-molecule inhibitors of retrograde transport strongly suppressed VACV multistep growth *in vitro* and reduced weight loss and clinical signs in an *in vivo* murine model of systemic poxviral disease. This work links cellular retrograde transport pathways with the morphogenesis of poxviruses and identifies a panel of novel inhibitors of poxvirus replication.

**IMPORTANCE** Cellular retrograde transport pathways traffic cargo from endosomes to the *trans*-Golgi network and are a key part of the intracellular membrane network. This work reveals that the prototypic poxvirus vaccinia virus (VACV) exploits cellular retrograde transport pathways to facilitate the wrapping of intracellular mature virions and therefore promote the production of extracellular virus. Inhibition of retrograde transport by small-molecule inhibitors reduced the replication of VACV in cell culture and alleviated disease in mice experimentally infected with VACV. This research provides fundamental new knowledge about the wrapping step of poxvirus morphogenesis, furthers our knowledge of the complex cellular retrograde pathways, and identifies a new group of antipoxvirus drugs.

## INTRODUCTION

Vaccinia virus (VACV) is the prototypic orthopoxvirus. It is a large double-stranded DNA virus with a complicated intracytoplasmic life cycle involving progression through four virion forms. The initial and most abundant virion type is the intracellular mature virion (IMV), which consists of a viral core surrounded by a single membrane. IMVs are fully infectious and can be released on cell lysis. Prior to lysis, a minority of the IMVs are wrapped in a double membrane to form intracellular enveloped virions (IEV), which have three membranes (reviewed in reference 1). The origin of both the IMV and IEV membranes is much debated (2). Once it is wrapped, the IEV is transported to the cell periphery, where its outer membrane fuses with the plasma membrane to form a double-wrapped cell-associated enveloped virion (CEV) on the surface of the cell. A selection of IEV-associated viral proteins becomes embedded in the plasma membrane of the cell as a result of this membrane fusion event. A proportion of the CEVs stimulates actin polymerization, resulting in the formation of actin tails (3, 4). Once CEVs detach from the cell surface, they are known as extracellular enveloped virions (EEVs) (1). CEVs and EEVs are few in number (often only 1% of the number of IMVs present in the cell) but crucial for the virus since they mediate spread between neighboring cells and to more distant cells *in vitro* and *in vivo* (5–7).

Previous research has suggested that VACV may use retrograde transport pathways as part of its complex virion morphogenesis. The viral IEV membrane proteins A33, A36, B5, and F13 were found to accumulate at the plasma membrane following disruption of clathrin-mediated endocytosis (8–10), leading to the hypothesis that clathrin-mediated endocytosis is the first step in retrograde transport of these proteins. In addition, two independent studies reported the very rapid transport of horseradish peroxidase (HRP) from the cell surface to the Golgi apparatus in VACV-infected cells (11, 12), indicating that the virus stimulates retrograde transport. More recently, endosome-to-Golgi retrograde transport pathway (EGRTP) proteins have been identified to be proviral host factors in two independent high-throughput small interfering RNA (siRNA) screens of VACV replication (13, 14). This evidence collectively supports the utilization of EGRTP by VACV; however, direct evidence is lacking.

EGRTPs are used by the cell to recycle cargo back to the *trans*-Golgi network (TGN) for reuse. The pathway can begin at early, late, or recycling endosomes, where cargo-sorting complexes, such as retromer, recognize and select cargo and sort it into vesicles, which are then transported to the TGN (15). Tethering factors, such as golgin-97 and the GARP (Golgi-associated retrograde protein) complex, tether the transport intermediates to the TGN and enable SNARE (soluble *N*-ethylmaleimide-sensitive fusion factor attachment receptor) complexes on the transport vesicles (vSNAREs) and target membrane (tSNAREs) to promote membrane fusion. Tethers are targeted to the TGN by small GTPases of the Rab family. Different cargoes use different combinations of cargo sorters, SNAREs, tethers, and GTPases (16). Well-characterized endogenous cargoes that are transported along retrograde pathways include mannose 6-phosphate receptor, TGN-46 (17), sphingolipids (18), and β1 integrin (19). In addition to cellular proteins, toxins also transit along retrograde vesicle pathways to access their site of action, including Shiga toxin, Shiga-like toxins 1 and 2, ricin, and cholera toxin (16). A number of viruses also utilize the pathway; for example, HIV uses it for trafficking of envelope glycoprotein (Env) (20), human papillomavirus 16 (HPV16) uses it for entry (21), and adeno-associated virus (AAV) uses it for transduction (22). Endosome-to-Golgi retrograde pathways are therefore key physiological transport routes that are also implicated in a number of pathogenic mechanisms.

This work outlines the role of EGRTP in VACV replication. The tetrameric tethering factor GARP was shown to be required for the progression of IMVs to EEVs and, more specifically, for the wrapping of IMVs to produce IEVs. A reduction in the amount of GARP markedly reduced the number of IEVs in the cell and caused cytoplasmic accumulation of aberrant structures containing IEV membrane proteins. The retrograde pathway used by VACV was found to be independent of retromer but was inhibited by small-molecule inhibitors of the EGRTP, including Retro-2. Further, intraperitoneal (i.p.) treatment of mice with Retro-2 prior to intranasal infection with VACV ameliorated weight loss and significantly reduced signs of disease, identifying EGRTP as a therapeutic target for preventing poxviral infection.

## MATERIALS AND METHODS

### Cells and viruses.

Dulbecco's modified Eagle's medium (DMEM; Life Technologies) containing 50 μg/ml streptomycin and 50 μg/ml penicillin (pen/strep; Sigma) with 10% fetal bovine serum (FBS; Life Technologies) was used to grow the following cell lines: rabbit kidney epithelial (RK-13) cells, African green monkey kidney epithelial (BS-C-1 and Vero) cells, and human cervix carcinoma epithelial (HeLa) cells. All cell lines were passaged regularly to maintain viability. Cells were washed in phosphate-buffered saline (PBS) and detached using 0.5% trypsin–EDTA (Life Technologies) before transfer to a fresh tissue culture flask. Wild-type (WT) and mutant (MU) mouse embryonic fibroblasts (MEFs) were provided by one of the authors (Thomas Schmitt-John) and were grown in pyruvate-free DMEM (Life Technologies) containing 50 μg/ml penicillin and 50 μg/ml streptomycin (Sigma) with 20% FBS (Life Technologies). MEFs were received as primary cells and were immortalized through their crisis stage by repeated 1:1 passages until cells became permissible for growth in tissue culture flasks. Once they were immortalized, MEFs were regularly passaged for maintenance as described above. All cells were grown at 37°C in an atmosphere with 5% CO_2_ and 95% humidity. VACV-A5-EGFP (VACV-EGFP) has been described previously (23). The viruses used in all experiments were purified by high-speed centrifugation through a 36% (wt/vol) sucrose cushion.

### siRNA transfection.

The siRNA transfection method has been described previously (14). siRNAs (Dharmacon/Thermo Scientific) were added to a final concentration of 25 nM, and the cells were incubated at 37°C in 5% CO_2_ for 48 h before further experimental steps were carried out. The sequences of the siRNAs used in this work are as follows: for VP16, GGGCGAAGUUGGACUCGUAUU; for RAB1A (SMARTpool), GAACAAUCACCUCCAGUUA, CAAUCAAGCUUCAAAUAUG, GGAAACCAGUGCUAAGAAU, and CAGCAUGAAUCCCGAAUAU; for VPS52 (SMARTpool), CGAAAGAGGCAGUAAGGAA, GAUCACACCCACAAUGAAA, GGCAAUGUCUCCACGGCAA, and CCAGAUGAUGGUUGAACAU; for VPS35 (SMARTpool), GAACAUAUUGCUACCAGUA, GAAAGAGCAUGAGUUGUUA, GUUGUAAACUGUAGGGAUG, and GAACAAAUUUGGUGCGCCU; and for VPS26 (SMARTpool), GCUAGAACACCAAGGAAUU, UAAAGUGACAAUAGUGAGA[, UGAGAUCGAUAUUGUUCUU, and CCACCUAUCCUGAUGUUAA.

### Western blotting.

Cells were washed twice with ice-cold PBS and then lysed in protein lysate buffer (50 mM Tris-Cl, pH 7.5, 100 mM NaCl, 1% [wt/vol] NP-40) and 1:7 protease inhibitor (Complete tablets; Roche) on ice for 30 min before storage at −70°C. After thawing, lysates were centrifuged at 16,100 × *g* for 5 min and the pellet was discarded. A bicinchoninic acid (BCA) protein assay kit (Thermo Scientific Pierce) was used to calculate protein concentrations according to the manufacturer's protocol, and equal amounts of protein were loaded for Western blotting, which was carried out as described previously (24). The primary antibodies used were rabbit anti-VPS52 (catalog no. ARP57644_P050; Aviva System Biology), mouse anti-actin (catalog no. 3700S; Cell Signaling), and anti-VPS35 (catalog no. Ab57632; Abcam), anti-VPS26 (catalog no. Ab23892; Abcam). The secondary antibodies were DyLight 680 and 800 (Cell Signaling).

### One-step and multistep growth curves.

One-step and multistep growth curves have been described previously (24, 25). Briefly, for one-step growth curves, cells were infected at a multiplicity of infection (MOI) of 5 PFU/cell for 1 h at 37°C and then washed with medium and incubated for the times indicated below. Supernatants were collected and centrifuged at low speed to remove the cell debris and then incubated with anti-L1 antibody (BEI Resources) for 1 h at 37°C in order to neutralize IMV particles. The titers of the virus present in the supernatants and cellular fraction were then determined on BS-C-1 cells. For the multistep growth curves, cells were infected at a low MOI (0.05 or 0.1 PFU/cell) for 1 h at 37°C. At the times indicated below, the supernatant and cellular fraction were combined by scraping the cells into the supernatant, and titers were determined by plaque assay on BS-C-1 cells.

### Immunofluorescence.

Cells to be examined for immunofluorescence were seeded on 20-mm by 20-mm coverslips at a density of 1.5 × 10^5^ cells for HeLa cells and 2 × 10^5^ cells per well for MEFs, and the coverslips were placed into six-well plates. The cells were fixed with 2 ml of neutral buffered formalin for 30 min, washed in PBS, permeabilized in 0.2% Triton X-100 for 5 min at room temperature, and washed 3 times in PBS. The cells were incubated with primary antibody at room temperature (RT) for 1 h in a humidity chamber. The coverslips were washed three times in PBS–2% FBS before addition of secondary antibody and fluorescently labeled phalloidin (Molecular Probes) diluted in PBS–2% FBS for 1 h at RT in a humidity chamber. After washing three times in PBS and once in distilled water, the coverslips were mounted in ProLong Gold mounting medium containing DAPI (4′,6-diamidino-2-phenylindole; Molecular Probes). The slides were examined on a Zeiss 710 confocal microscope. The primary antibodies used were sheep anti-TGN46 (catalog no. AHP500; AbD Serotec), mouse anti-B5 (catalog no. NR-556; BEI Resources), and rat anti-F13. Secondary antibodies were sourced from Life Technologies (Alexa Fluor 488-conjugated goat anti-rabbit immunoglobulin, Alexa Fluor 568-conjugated goat anti-rabbit immunoglobulin, Alexa Fluor 568-conjugated goat anti-mouse immunoglobulin, and Alex Fluor 594-conjugated donkey anti-sheep immunoglobulin).

### Imaris image analysis.

Three-dimensional (3D) image analysis was carried out using Imaris image analysis software (Imaris, version 8.2; Bitplane AG, Switzerland). Two-dimensional confocal z-stacks were acquired on a Zeiss 710 microscope and rendered into three dimensions. The ImarisCell module was then used to segment the nucleus, cell, and B5-labeled vesicles. Vesicles were exported to spots and filtered for enhanced green fluorescent protein (EGFP) colocalization to identify B5-labeled structures with a core center.

### Pharmacological inhibition of retrograde transport *in vitro*.

Retro-1, VP-184, Retro-2, and Retro-2.1 were synthesized in-house (26, 27) and resuspended in dimethyl sulfoxide (DMSO) before serial dilution to a range of concentrations in FBS-free, antibiotic-free DMEM. Appropriate dilutions were added to a 6-well plate seeded with HeLa cells, and the plates were incubated for 2 h at 37°C in 5% CO_2_ before infection with VACV-EGFP for the length of time indicated below. The drugs remained present throughout infection and postinfection incubation. VP-184 is a Retro-1 analogue which gives increased protection against Shiga toxin and Simkania negevensis (28), and Retro-2.1 is an *N*-methyldihydroquinazolinone derivative of Retro-2 with enhanced efficacy against Shiga toxin (26).

### Retro-2 intraperitoneal administration.

Retro-2 was resuspended in DMSO to a concentration of 66.6 mg/ml (210 mM). Groups of 6 mice were injected intraperitoneally with 270 μl of PBS supplemented with 30 μl (10%, vol/vol) DMSO alone (control) or 30 μl Retro-2.

### Intranasal infection of mice.

BALB/c mice (female; age, 6 to 8 weeks) were inoculated with VACV-WR, which had been purified by sedimentation through a sucrose cushion. The mice were lightly anesthetized with isoflurane before 1 × 10^4^ PFU VACV-WR diluted in 20 μl PBS was inoculated into the nostril. The mice were monitored daily for weight loss and scored for clinical signs of disease (hair ruffling, hunched back, reduced mobility). All animal experiments were carried out under a UK Home Office license and assessed by the local Animal Ethics and Welfare Committee.

### Titration of virus from mouse lungs.

Lungs from each mouse were placed in a safe-lock Eppendorf tube (Eppendorf) with one 5-mm stainless steel bead (Qiagen) and 1 ml DMEM supplemented with 2.5% FBS and pen/strep antibiotics. The tubes were arranged evenly and securely in a Qiagen TissueLyser II tissue disrupter, and the lung tissue was thoroughly disrupted by high-speed shaking (two pulses of 28,000 Hz for 2 min). Samples were then transferred to fresh standard Eppendorf tubes and spun on a tabletop centrifuge for 5 min at 3,000 rpm. The supernatants were titrated by plaque assay on BS-C-1 cells.

## RESULTS

### The GARP complex is required for multistep replication of VACV.

**FIG 1:**
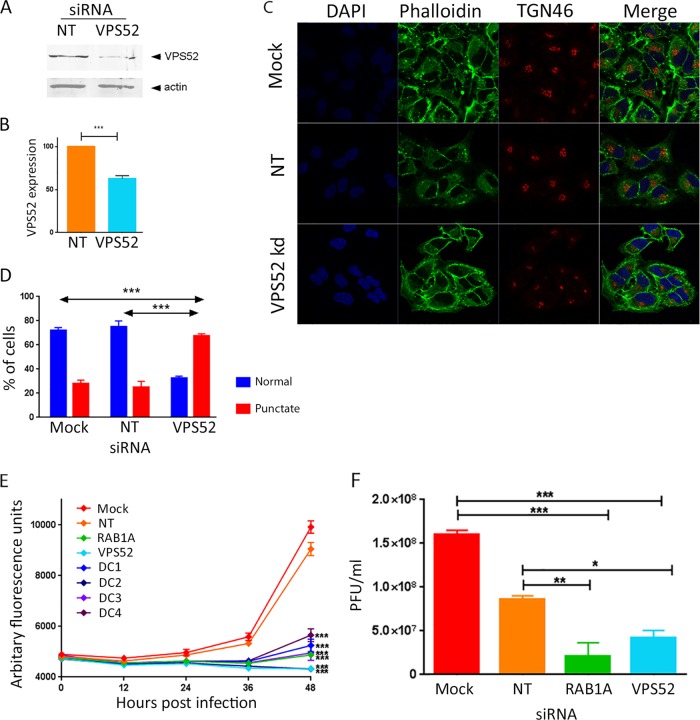
Reduced expression of VPS52 impairs multistep growth of VACV. (A) HeLa cells were transfected with nontargeting siRNA (NT) or a SMARTpool of four siRNAs targeting VPS52. After 48 h, cells were harvested and proteins were separated using SDS-PAGE, probed with antibody raised against VPS52 or actin, and visualized using direct infrared fluorescence (LI-COR) in an Odyssey scanner. (B) The levels of VPS52 relative to those of actin were quantified. Data represent the averages from three biological replicates. Error bars are SEMs. ***, *P* < 0.001 by two-tailed Student's *t* test. (C) HeLa cells were mock transfected or transfected with nontargeting siRNA or siRNA targeting VPS52. After 48 h, cells were fixed and labeled with antibody raised against TGN46 and phalloidin. (D) The percentage of cells with a normal punctate TGN46 distribution was calculated. ***, *P* < 0.001 by two-tailed Student's *t* test. (E) HeLa cells were transfected with a VPS52 siRNA SMARTpool (VPS52) and the four individual deconvoluted VPS52 siRNAs (DC1 to DC4). Negative controls included mock-transfected cells and cells transfected with nontargeting siRNA. siRNA targeting a known proviral host factor (RAB1A) was used as the positive control. After 48 h, cells were infected with VACV-EGFP at 0.1 PFU/cell, and fluorescence levels were measured over the following 48 h. The data represent those from six technical replicates and are representative of those from three biological replicates. Error bars represent SEMs. Results were analyzed with a one-way analysis of variance with multiple comparisons at 48 h postinfection. ***, *P* < 0.001. (F) HeLa cells were mock transfected or transfected with nontargeting siRNA or siRNA targeting VPS52 or RAB1A. After 48 h, cells were infected with VACV-EGFP at 0.1 PFU/cell, and at 48 h p.i., cells and supernatant were collected and virus titers were determined. Data were analyzed with a one-way analysis of variance with multiple comparisons. ***, *P* < 0.001; **, *P* < 0.01; *, *P* < 0.05.

The GARP complex is a tetramer composed of four proteins (VPS51, VPS52, VPS53, and VPS54). It localizes to the TGN, where it acts as a tethering factor, receiving transport vesicles that are traveling back from endosomes. Knockdown of a single component of the tetramer destabilizes the complex and results in the rapid decay of all four components and impaired retrograde transport (29, 30). Two subunits of the GARP complex (VPS54 and VPS52) were identified to be strong proviral host factors in independent siRNA screens of VACV replication (13, 14). In order to investigate this further, the levels of VPS52 protein were depleted using siRNA and the impact on VACV multistep replication was measured. HeLa cells were mock transfected or transfected with either a nontargeting control siRNA (targeting herpes simplex virus 1 [HSV-1] protein VP16) or siRNA targeting VPS52. A siRNA SMARTpool containing four different siRNAs, all of which targeted VPS52, was used to enhance the magnitude and specificity of protein knockdown. After 48 h of transfection, the cells were harvested and the level of VPS52 in the cell lysates was compared using Western blotting. In cells transfected with VPS52-targeting siRNA, the level of VPS52 was consistently reduced to approximately 60% of the level in cells treated with nontargeting control siRNA (Fig. 1A and B), confirming the efficacy of the SMARTpool.

The impact of VPS52 reduction on the functioning of retrograde transport pathways was assessed by examining the location of the endogenous TGN marker TGN46, which is known to be transported on retrograde pathways (17). HeLa cells were mock transfected or transfected with siRNA targeting VPS52 or nontargeting control siRNA and, after 48 h, fixed and labeled with antibody raised against TGN46 as well as with fluorescent phalloidin to outline the cytoskeleton. In the mock-transfected and nontargeting siRNA-transfected cells, TGN46 displayed a classic perinuclear location, consistent with the location of the *trans*-Golgi network. However, in cells transfected with siRNA targeting VPS52, the localization of TGN46 was altered to a much more punctate, juxtanuclear distribution (Fig. 1C), consistent with the findings presented in previous reports (17). When multiple cells were scored for normal or collapsed TGN46 localization, a highly statistically significant disruption of TGN46 localization, indicative of the disruption of EGRTP function, was evident in response to the depletion of VPS52 (Fig. 1D).

The impact of VPS52 on VACV replication and spread was then examined using VACV-A5-EGFP, a VACV strain which has the A5 viral core protein tagged with EGFP, thereby enabling virus growth to be estimated by measuring fluorescence levels (14, 23). HeLa cells were mock transfected or transfected with nontargeting siRNA (a negative control); siRNA targeting RAB1A (a positive control), which is required for efficient VACV replication (25); or the VPS52 siRNA SMARTpool. In addition, the VPS52 SMARTpool was deconvoluted to its four constituent siRNAs, and each was tested individually. After 48 h, the cells were infected with VACV-A5-EGFP at a low MOI of 0.1 PFU/cell, and fluorescence was measured at 12-h intervals for a further 48 h, allowing multiple rounds of virus replication to occur (Fig. 1E). Fluorescence levels increased over time, as expected, and were comparable in the negative-control samples (mock and VP16 transfected) but significantly reduced at 36 and 48 h postinfection (p.i.) in the wells treated with positive-control siRNA (RAB1A), the VPS52 SMARTpool, and all four of the deconvoluted VPS52 siRNAs.

In order to determine directly the effect of depletion of VPS52 on infectious virion production, the experiment was repeated and VACV replication was measured using virus titration (Fig. 1F). After 48 h there was an average of a 1-log_10_ reduction in the amount of virus produced in the positive-control samples depleted of RAB1A, consistent with previous findings (25). In cells depleted of VPS52, there was a smaller but reproducible and statistically significant (*P* < 0.05) reduction of 0.5 log_10_ in the amount of virus detected compared to the amount in the negative-control samples transfected with nontargeting siRNA. Overall, these results substantiate previously reported evidence from the high-throughput siRNA screens that VPS52 is required for the efficient multicycle growth of VACV.

### Genetically induced destabilization of GARP impairs VACV replication.

Depletion of GARP using siRNA resulted in a consistent and significant reduction of VACV replication in HeLa cells, but one that was less marked than that seen in response to depletion of other proviral host factors previously investigated (14). A possible explanation for this was the limited efficacy of the VPS52 siRNA treatment. As seen in Fig. 1D, 25% of the population of cells transfected with VPS52-targeted siRNA showed normally distributed TGN46, suggesting that the protein knockdown was insufficient in all cells to fully ablate EGRTP function. In order to achieve a uniform reduction in EGRTP function and to validate the siRNA knockdown findings using an alternative method, we used a mouse genetic mutant with a hypomorphic mutation in the VPS54 subunit of GARP (30). In this mouse, a single missense mutation converting codon 967 of the *vps54* gene from leucine to glutamine results in an unstable VPS54 protein with a shortened half-life (29). This results in less overall GARP and a partial loss of function, as seen in slower transport and mislocalization of the retrograde-dependent cholera toxin B subunit (31). Wild-type (WT) and mutant (MU) primary mouse embryonic fibroblasts (MEFs) were isolated and immortalized through serial passage. In the following experiments, two independently derived WT cell lines and two MU cell lines were included to reduce the likelihood that off-target mutations produced during immortalization were responsible for the phenotype seen.

**FIG 2:**
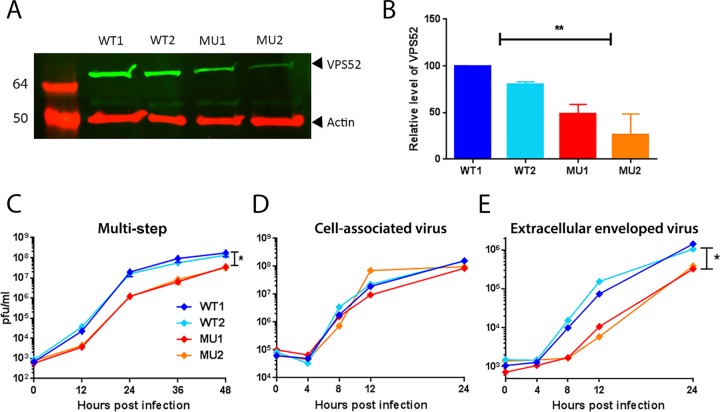
Mutation of the GARP complex reduces VACV replication in a multistep growth curve. (A) Protein lysates were collected from two independently immortalized lines of wild-type MEFs (WT1 and WT2) and mutated MEFs (MU1 and MU2), separated using SDS-PAGE, probed with antibody raised against VPS52 or actin, and visualized using direct infrared fluorescence (LI-COR) in an Odyssey scanner. Numbers on the left are molecular sizes (in kilodaltons). (B) The levels of VPS52 relative to those of actin were quantified. Data represent the averages from three biological replicates. Error bars are SEMs. **, *P* < 0.01, one-way analysis of variance with multiple comparisons. (C) Multistep growth curve. WT or MU MEFs were infected with VACV-WR at 0.01 PFU/cell. Cells were harvested at the indicated times p.i., and total virus levels were determined by plaque assay on BS-C-1 cells. The graph shows the mean titer from three biological replicates. Error bars indicate SEMs. *, *P* < 0.05, one-way analysis of variance with multiple comparisons at 48 h p.i. (D and E) One-step growth curve. WT or MU MEFs were infected with VACV-WR at 5 PFU/cell. At the indicated times p.i., cells (D) and supernatant (E) were collected and virus titers were determined by plaque assay on BS-C-1 cells. The graphs show the mean titers from three biological replicates. Error bars indicate SEMs. *, *P* < 0.05, one-way analysis of variance with multiple comparisons.

To confirm that the hypomorphic mutation destabilizes the GARP complex, cell lysates of WT and MU immortalized MEFs were collected and the levels of VPS52 were compared using Western blotting. As shown in Fig. 2A and B, the levels of VPS52 protein were significantly reduced in the mutant cells by an average of 52%. A VACV multistep growth curve was then carried out on the two WT cell lines and the two MU cell lines, and the amount of virus present was determined by titration (Fig. 2C). A clear difference in virus titer between the WT and MU cell lines was apparent from 12 h p.i., with an approximately 1-log_10_ reduction in the amount of virus being present in both MU cell lines at 12, 24, and 36 h p.i., and the amount narrowed to 0.5 log_10_ at 48 h p.i. as virus production plateaued. This result confirms by an independent method and in a second cell type that GARP is required for optimal multistep VACV replication.

### GARP is required for production of IEVs and CEVs but not IMVs.

In order to examine the impact of GARP depletion on the different virion forms produced during VACV replication, we carried out a one-step growth curve on WT and MU MEFs, infecting the cells at a high MOI (5 PFU/cell) and measuring virus production over the following 24 h. The titers of virus present in the cells and the supernatant were determined separately. Neutralizing antibody to IMVs was added to the supernatant fraction prior to titration to ensure that only the titers of EEVs that had been released from the cell were determined. No significant difference in the amount of virus produced in the cellular fraction (which consists almost entirely of IMVs) was detected at any time point (Fig. 2D), suggesting that initial stages of virus production were unaffected by GARP depletion. However, a reduction in virus titer in the supernatant fraction of up to 1 log_10_ in the MU cells compared to the titers in the WT cells was detected at 8, 12, and 24 h p.i. (Fig. 2E), indicating that fewer EEVs were produced from cells lacking normal levels of GARP.

**FIG 3:**
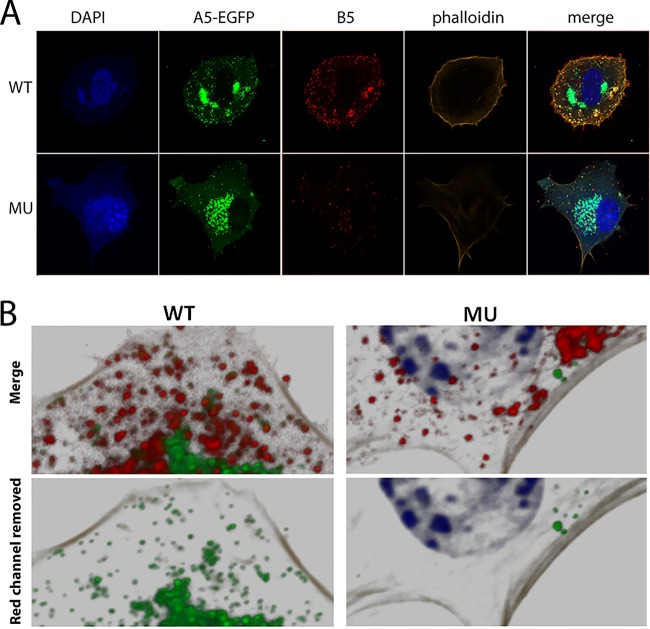
Mutation of the GARP complex impairs production of IEVs. (A) WT and MU MEFs were infected with VACV-A5-EGFP (green) at 5 PFU/cell for 8 h and then fixed and labeled with anti-B5 antibody (red), phalloidin (orange), and DAPI (blue). Images are representative confocal sections acquired with a Zeiss LSM 710 confocal microscope. (B) 3D reconstruction of z-stack confocal series of WT and MU MEFs infected with VACV-EGFP. (Top) DAPI (blue), VACV cores (green), B5 (red), and actin (phalloidin, gray); (bottom) the same images from the top panels with the red (B5) channel removed.

**FIG 4:**
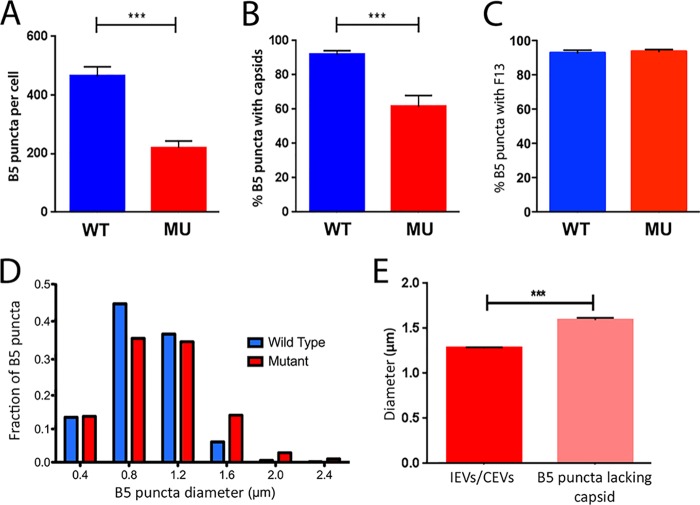
Mutation of the GARP complex disrupts the wrapping of IMVs, resulting in aberrant B5 accumulations. Imaris image analysis software (Bitplane) was used to quantitate the number of B5-labeled puncta in each cell (A), the percentage of B5-labeled puncta in a cell which colocalized with an EGFP-labeled core (B), the percentage of B5 puncta which colocalized with F13 (C), and the diameter of B5-labeled puncta in WT and MU MEFs (D). Data represent the averages and SEMs from an analysis of 49 cells (24 WT and 25 MU cells) from three independent experiments. ***, *P* < 0.001 by two-tailed Student's *t* test. (E) The diameter of B5-labeled puncta in MU MEFs was compared. The diameter of normal B5-labeled puncta which were associated with an EGFP-labeled core and the diameter of abnormal B5-labeled puncta with no associated core were compared. Data represents the averages and SEMs from an analysis of 25 MU cells from three independent experiments. ***, *P* < 0.001 by two-tailed Student's *t* test.

To identify the point at which EEV production was impacted by reduced levels of GARP, we assessed and compared the number of IEVs and CEVs present in the WT and MU MEFs using immunolabeling and confocal fluorescence microscopy. MEFs were infected with VACV-A5-EGFP at a high MOI of 5 PFU/cell and, after 8 h, fixed, permeabilized, and labeled with antibody targeted to the IEV/CEV membrane protein B5 (32). Single optical slices taken through infected WT cells showed that the EGFP-tagged A5 protein was distributed in the expected pattern of large perinuclear viral assembly factories as well as in much smaller dispersed cytoplasmic puncta representing individual virus cores (Fig. 3A, top). Many, but not all, of these cores also stained positive for B5, thereby identifying them as IEVs/CEVs. However, this pattern altered in GARP mutant cells, as while there were still abundant peripherally dispersed viral cores (consistent with the unaltered titers of cell-associated virus), there were noticeably fewer puncta of B5 staining (Fig. 3A, bottom). Furthermore, high-magnification analysis of viral core and B5 localization showed that while most B5 puncta colocalized with the VACV-A5-EGFP fluorescence in WT cells (indicating that the foci were indeed IEV/CEVs), the MU cells contained many more B5 foci that lacked an associated core (Fig. 3B). Twenty-five WT and 24 MU cells from three independent experiments were analyzed by taking z-stacked images throughout the depth of the cells, reconstructing them as a 3D image, and analyzing the data using Imaris image analysis software to quantify the number of B5-labeled puncta. WT cells contained, on average, over 450 puncta per cell, but this was more than 2-fold lower in the MU cells (Fig. 4A). Analysis of the number of B5 puncta that coincided with EGFP fluorescence also confirmed a statistically significant drop in the proportion associated with cores, from over 90% in WT cells to about 60% in MU cells (Fig. 4B). However, when cells were costained for B5 and another IEV/CEV membrane protein, F13, there was no decrease in the level of colocalization in the MU cells, with a high proportion (over 90%) of vesicles being double positive for both IEV/CEV membrane proteins in WT and MU cells (Fig. 4C).

Further differences between B5-labeled structures in the cytoplasm of WT and MU MEFs were also evident, in that B5-labeled puncta in the MU cells showed more variation in size and had a small but statistically significant increase in their average diameter compared with IEVs/CEVs in the WT cells, from 0.96 μm to 1.08 μm. Frequency analysis of the data showed that this reflected an increase in the population of puncta with diameters of ≥1.6 μm in the MU cells (Fig. 4D). The two populations of B5 puncta in the MU cells were compared. The abnormal B5-labeled structures lacking an associated core were found to be significantly larger (125%) than the puncta associated with a core (IEVs/CEVs) (Fig. 4E). These results reveal that IMV wrapping is disrupted when the GARP complex is dysfunctional, resulting in accumulation in the cytoplasm of aberrant large B5-labeled puncta lacking an internal core.

### VACV replication is independent of the retromer cargo-sorting protein complex.

**FIG 5:**
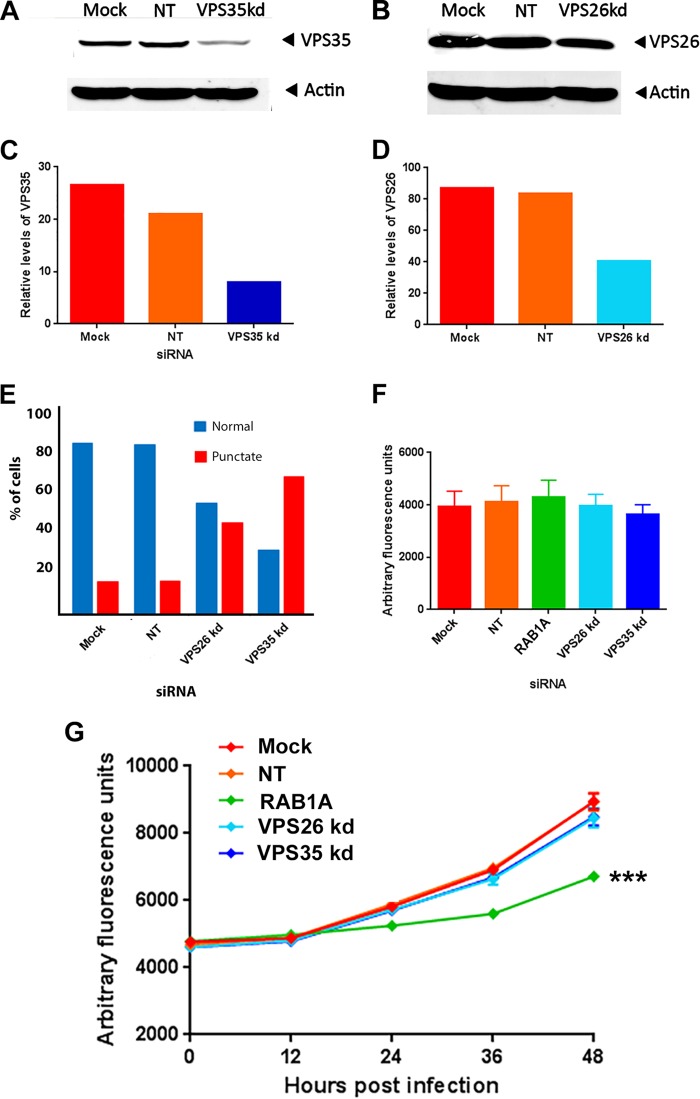
VACV morphogenesis is independent of the retromer complex. HeLa cells were mock transfected or transfected with nontargeting siRNA (NT) or siRNA targeting VPS35 (A) or VPS26 (B). After 48 h, cells were harvested and proteins were separated using SDS-PAGE, probed with antibody raised against VPS35 or actin, and visualized using direct infrared fluorescence (LI-COR) in an Odyssey scanner. The levels of VPS35 (C) and VPS26 (D) relative to those of actin were quantified. (E) HeLa cells were mock transfected or transfected with nontargeting siRNA or siRNA targeting VPS26 or VPS35. After 48 h, cells were fixed and labeled with antibody raised against TGN46 and phalloidin. The percentage of cells with a normal punctate TGN46 distribution was analyzed. (F and G) HeLa cells were mock transfected or transfected with nontargeting siRNA or siRNA targeting VPS35, VPS26, or RAB1A (positive control). (G) After 48 h, cells were infected with VACV-EGFP at 0.1 PFU/cell and fluorescence was measured at the indicated times. (F) At 48 h p.i., cell death was measured using CellTiter-Blue and is presented as arbitrary fluorescence units. The data represent those from six technical replicates, and error bars represent SEMs. Results were analyzed by Student's *t* test. ***, *P* < 0.001. kd, knockdown.

In order to define the retrograde transport pathway components involved in VACV replication, we investigated the role of the retromer complex. Retromer is a well-characterized key component of endosome-to-Golgi retrograde pathways. It is located in the endosomal membrane and acts as a cargo sorter, binding to cargo in the lumen that is destined for retrograde trafficking and facilitating the formation and budding off of retrograde transport vesicles (33). We separately targeted two subunits of retromer (VPS35 and VPS26) using siRNA transfected into HeLa cells. Western blotting indicated successful knockdown of protein expression to levels less than 50% of those detected in cells transfected with nontargeting siRNA (Fig. 5A to D). To confirm that retrograde transport was disrupted in these cells, the location of TGN46 was examined. Cells with reduced levels of VPS35 or VPS26 revealed an abnormal localization of TGN46, confirming functional disruption (Fig. 5E). The efficacy of multistep VACV growth in cells with a knockdown in expression of either the VPS26 or the VPS35 subunit of the retromer complex was then assessed by infecting the siRNA-transfected cells with VACV-A5-EGFP at a low MOI of 0.1 PFU/cell and measuring the fluorescence every 12 h as a marker of virus replication (Fig. 5G). The positive-control siRNA which targets RAB1A, a known proviral host factor, reduced the fluorescence levels by over 25% at 48 h p.i.; however, a reduction of either retromer subunit had no significant effect on VACV replication compared to that in mock-transfected cells or cells transfected with the control nontargeting siRNA. No significant difference in cell death was detected in the different treatment groups after 48 h of virus infection, proving that the siRNA treatments were not toxic (Fig. 5F). This result suggests that VACV uses a nonclassical, retromer-independent retrograde trafficking pathway.

### VACV is susceptible to pharmacological inhibition of retrograde transport pathways.

Toxins such as ricin, Shiga toxin, and cholera toxin use EGRTP to escape the endosomal compartment and travel through the cytosol of the cell (16). A high-throughput screen of a small-molecule library in a ricin cytotoxicity assay identified potent pharmacological inhibitors of EGRTP, including Retro-1 and Retro-2, two compounds with a heterocyclic structure incorporating a central benzodiazepine or imine moiety (34). The mechanisms of function of Retro-1 and Retro-2 are unknown; however, they both cause a rapid redistribution of the SNARE protein syntaxin 5 (22, 34). The effect of both drugs is highly specific to retrograde endosome-to-TGN pathways; neither drug interferes with anterograde transport along the biosynthetic/secretory pathway from the endoplasmic reticulum via the Golgi apparatus and TGN to the plasma membrane or with endocytosis, recycling from the early endosome back to the plasma membrane or trafficking to late endosomes and the lysosome (31, 34). This specificity of action makes Retro-1 and -2 powerful tools when investigating retrograde transport.

**FIG 6:**
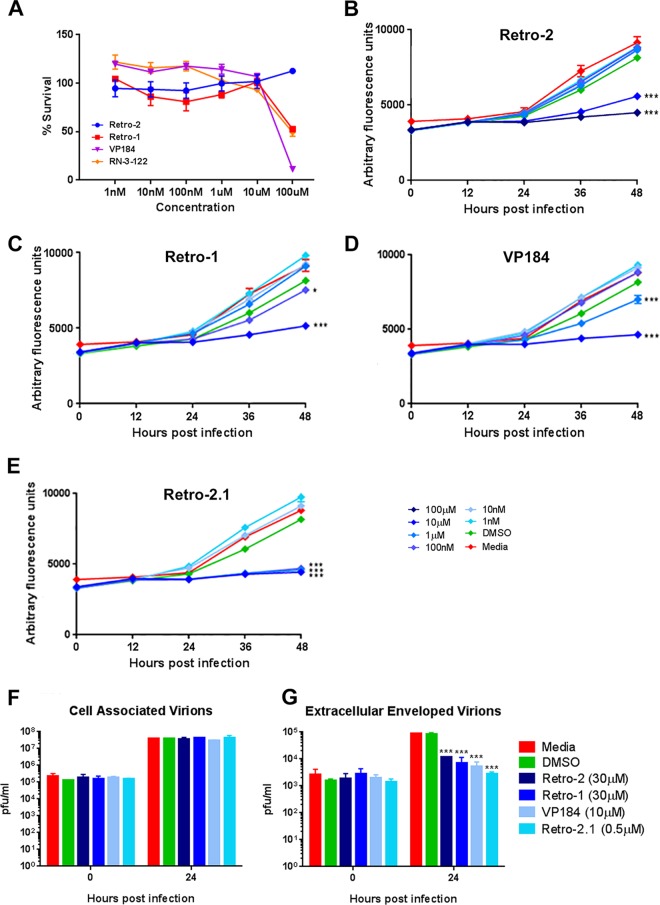
Inhibition of retrograde transport with small-molecule inhibitors reduces IEV production. (A) HeLa cells were treated with various concentrations of drug, as indicated, and infected with VACV-EGFP at 0.1 PFU/cell. After 48 h, cell death was measured using CellTiter-Blue. Cell death is expressed as a percentage of the survival of infected cells treated with medium alone. Data are averages and SEMs from six technical replicates. (B to E) HeLa cells were treated with various concentrations of the indicated drug and infected with VACV-EGFP at 0.1 PFU/cell. Fluorescence was measured at the indicated time points. Data are average and SEMs from six technical replicates and were analyzed using a one-way analysis of variance with multiple comparisons of the data at 48 h postinfection. ***, *P* < 0.001. (G and H) One-step growth curve. HeLa cells were treated with the compounds indicated and infected with VACV-WR at 5 PFU/cell. At 0 and 24 h p.i., cells (F) and supernatants (G) were collected and virus titers were determined by plaque assay on BS-C-1 cells. ***, *P* < 0.001, one-way analysis of variance with multiple comparisons of the data at 48 h postinfection.

The effect of Retro-1 and Retro-2 on VACV multistep replication was investigated. In addition, two related drugs, VP-184 and Retro-2.1, were also tested. VP-184 is closely related to Retro-1 (28), while Retro-2.1 is a novel, highly potent derivative of Retro-2 (26). HeLa cells were pretreated for 1 h with various concentrations of drug and then infected at a low MOI (0.05 PFU/cell) with VACV-A5-EGFP, and fluorescence was measured over the following 48 h. At 48 h p.i., cell viability was measured using CellTiter-Blue (Promega) and compared to the viability of cells infected and treated with medium alone. Increased levels of cell death were detected only at the highest concentration (100 μM) of Retro-1, VP-184, and Retro-2.1 tested (Fig. 6A). No cell death was present even at a 100 μM Retro-2 concentration, indicating a very low toxic impact of the drug. EGFP fluorescence measurement revealed a strong inhibitory effect of Retro-1, VP-184, Retro-2, and Retro-2.1 on VACV growth (Fig. 6B to E) compared to the growth of the controls treated with medium or DMSO. This suggests that endosome-to-Golgi apparatus trafficking is required for VACV multistep replication.

The concentrations of Retro-1 (1 μM and 10 μM) and Retro-2 (10 μM and 100 μM) that showed efficacy against VACV replication are comparable to the concentration (25 μM) used to block the activity of ricin and Shiga toxin (34). When comparing the effective concentrations of Retro-2 and Retro-2.1, we found that the more potent derivative drug, Retro-2.1, displayed a 100-fold increase in efficacy against VACV, with over 90% inhibition of virus replication being found at a concentration of just 100 nM. This finding is similar to that of previously reported work which found Retro-2.1 to be 100-fold more potent at preventing AAV transduction (22) and 500- to 1,000-fold more potent at blocking Shiga toxin and ricin toxicity (27).

To determine the stage of the VACV life cycle that is targeted by the four retrograde transport inhibitors, a one-step growth curve was carried out in the presence of drug, DMSO, or medium alone, and the cell-associated virus titer was determined separately from the titer of virus present in the supernatant after 24 h of infection. There was no detectable difference in the titer of cell-associated virus, with a greater than 2-log_10_ increase in the amount of virus present in all samples being found after 24 h. In contrast, the amount of EEV in the supernatant increased by over 1.5 log_10_ in the control samples treated with medium alone or carrier (DMSO) but less than 1 log_10_ in the samples treated with any of the four drugs (Fig. 6F and G). These results are strikingly similar to those obtained from one-step growth curves of MEFs expressing a GARP mutant (Fig. 2D and E) and provide compelling evidence that VACV uses EGRTP to facilitate virion morphogenesis.

### Retro-2 treatment alleviates systemic disease in mice infected with VACV.

**FIG 7:**
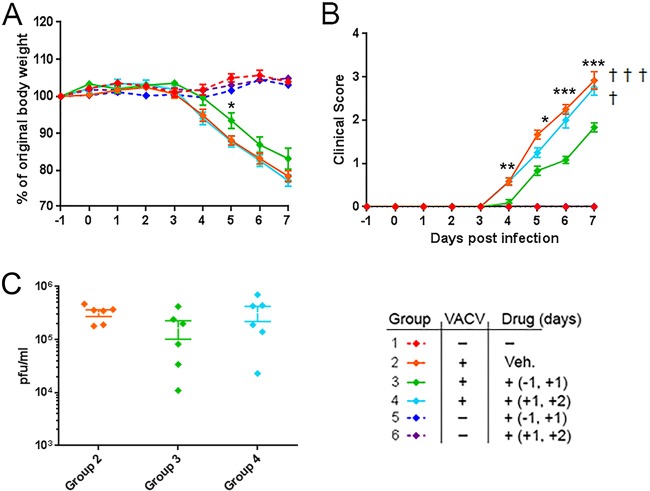
Retro-2 treatment prior to VACV infection ameliorates weight loss and clinical signs of disease in mice. BALB/c female mice were randomly assigned to 6 groups of 6 mice each. Mice in group 1 were untreated and uninfected controls. Mice in group 2 were infected with 1 × 10^4^ PFU of purified VACV-WR intranasally (i.n.) on day 0 and sham treated with a 300-μl i.p. injection of the 10% (vol/vol) DMSO in PBS vehicle (Veh.) on days +1 and +2 p.i. Mice in group 3 were treated with 100 mg/kg Retro-2 i.p. on days −1 and +1 and inoculated with 1 × 10^4^ PFU of purified VACV-WR i.n. on day 0. Mice in group 4 were infected i.n. with 1 × 10^4^ PFU VACV-WR and treated with 100 mg/kg Retro-2 i.p. on days +1 and +2 p.i. Mice in group 5 were treated with 100 mg/kg Retro-2 i.p. on days −1 and +1 p.i. Mice in group 6 were treated with 100 mg/kg Retro-2 i.p. on days +1 and +2 p.i. Mice in both groups 5 and 6 were left uninfected. All mice were monitored daily for weight change (A) and clinical signs of disease (B). (C) At the termination of the experiment (day 7), the viral loads in the lungs of mice in groups 2, 3, and 4 were determined by plaque assay. Statistical analysis was carried out using Student's *t* test to compare groups 2 and 3. *, *P* ≤ 0.05; **, *P* ≤ 0.01; ***, *P* ≤ 0.001. Each animal that reached the humane endpoint and then euthanized is indicated (†).

Inhibition of EGRTP *in vitro* strongly inhibited the production of EEVs. *In vivo* EEVs are the virion form responsible for the systemic spread of VACV around the body (5, 7). We therefore investigated whether pharmacological inhibition of EGRTP influenced disease in mice experimentally infected intranasally with VACV. Mice were injected intraperitoneally with 100 mg of Retro-2 per kg of body weight on two occasions. One group received the drug 24 h before and 24 h after infection with 1 × 10^4^ PFU VACV, while a second group received the drug 24 h and 48 h after infection with VACV. Control groups received no drug and no virus, drug alone, or virus inoculation plus intraperitoneal (i.p.) injection of vehicle alone (DMSO). Mice given i.p. drug but no virus consistently gained weight across the experiment and were indistinguishable from the negative-control group of mice given no drug or virus (Fig. 7A; compare groups 1, 5, and 6). This confirms previous reports of the excellent safety profile of Retro-2 after i.p. injection in mice (34–36). As expected, mice infected with VACV and treated with DMSO lost weight rapidly between day 4 and day 7 of the experiment and exhibited ruffled fur, a hunched posture, and reduced activity. Three out of the 6 mice in this group were euthanized due to the severity of clinical signs. Mice infected with VACV and treated with Retro-2 at 24 and 48 h postinfection lost weight just as rapidly as the untreated infected mice, and one mouse was euthanized. However, mice treated with Retro-2 24 h prior to and 24 h after VACV infection lost less weight (*P* = 0.05) and exhibited significantly less severe clinical signs of disease (*P* < 0.01) (Fig. 7A and B; compare group 3 with groups 2 and 4). None of the mice in this group reached the humane endpoint requiring euthanasia, revealing a protective effect of Retro-2 on VACV-induced disease in mice. The amount of virus in the lungs of each mouse in groups 2, 3, and 4 was determined (Fig. 7C). The average number of PFU of VACV in the lungs of mice in group 3 was lower than that in the lungs of mice in groups 2 and 4; however, this reduction did not reach the level of statistical significance (group 2 versus group 3, *P* = 0.07, Student's *t* test).

## DISCUSSION

This work outlines the reliance of VACV on cellular endosome-to-Golgi retrograde transport pathways in order to produce the wrapped IEV form. Retrograde pathways were inhibited by three different mechanisms (siRNA knockdown, a genetic hypomorphic mutation, and pharmacological inhibition), all of which resulted in a significant reduction in VACV multistep replication. Closer analysis revealed that the levels of IMV were unaffected by a reduction of the level of expression of the GARP complex; however, there was a drop in the number of IEVs/CEVs accompanied by aberrant accumulations of B5, indicating that the wrapping of IMVs had been disrupted. Wrapping of IMVs in an additional two layers of membrane is a poorly understood but crucial process in the life cycle of VACV and other poxviruses (2). The site of wrapping is debated, with evidence suggesting either the TGN (12) or endosome (11). The viral proteins A27, F13, and B5 are necessary for wrapping to occur, but the exact function of each of these proteins in the process is unclear (37–39). The identification of retrograde pathways and, specifically, the GARP complex as a crucial component of this process marks a decisive step forward in our understanding of this unique stage of poxvirus morphogenesis.

The GARP complex was targeted in our studies on the basis of results from two siRNA screens. A screen from our laboratory identified the VPS52 subunit to be a strong proviral host factor (14), and a second independent screen identified the VPS54 subunit (13). GARP is a member of the CATCHR (complexes associated with tethering containing helical rods) family of multisubunit tethering complexes, which are large proteins or protein complexes that establish long-range interactions between transport vesicles and their target compartment. GARP is localized to the TGN, where it facilitates tethering and, ultimately, the fusion of transport vesicles traveling in a retrograde direction from endosomes to the TGN (40). Cargoes which require GARP for transport to the TGN include receptors for lysosomal hydrolase precursors, such as the mannose 6-phosphate receptors, the TGN-resident protein TGN46 (17), and sphingolipids (18). Our work supports a model whereby the GARP complex tethers transport vesicles containing viral membrane proteins as they are recycled back from the plasma membrane to the TGN via retrograde transport pathways for reuse. VACV proteins embedded in the outer membrane of IEVs, including B5 and F13, remain on the surface of the cell after the IEV fuses with the plasma membrane during virion exit from the cell (1), and green fluorescent protein-tagged overexpressed B5 or F13 chimeras have been shown to relocate from the cell surface to a juxtanuclear location (8–10), suggesting retrograde transport of these crucial membrane proteins. In this model, the abnormal B5 puncta that lack an internal core that were identified in the MEFs with a mutated GARP complex would represent returning transport intermediates which were unable to dock with the site of IMV wrapping and IEV production. This model would support the hypothesis that the wrapping membranes originate from the TGN. However, we cannot rule out an alternative model whereby the GARP complex is needed for the recycling and correct localization of an as-yet-unidentified host protein(s) which is required for the wrapping process. In this model, the abnormal B5 accumulations would represent IEV membranes which failed to be successfully wrapped around the IMV, somewhat analogous to HSV-1 L particles (41).

In order to characterize the abnormal B5 accumulations, we compared the location of B5 and another IEV/CEV protein, F13, in WT and MU MEFs. F13 colocalized with B5 in both cell types, suggesting that the abnormal B5 puncta in the MU MEFs are likely related to IEV wrapping membranes rather than accumulations of individual mislocalized viral proteins.

We have uncovered evidence that VACV uses a nonclassical route of retrograde transport that is independent of the protein complex retromer. Previous work characterized two main retrograde pathways, with the first one being from early endosomes to the TGN (sometimes via recycling endosomes) and the second once being from late endosomes to the TGN. Each pathway involves a specific set of vSNARES, tSNARES, tethers, and GTPases; however, both rely on retromer for initial sorting of the endosomal cargo (16, 42). Retromer therefore plays a key role in both these pathways and is required for retrograde transport of many endogenous proteins as well as the correct localization of the HIV Env protein (20), the entry of HPV16 (21), and the transport of Shiga toxin (43, 44). Retromer is not, however, required for the retrograde pathways involved in AAV transduction (22) or VACV IEV formation (this work). Our results therefore confirm the existence of a retromer-independent retrograde transport pathway distinct from the two classical pathways previously described and highlights the complexity of cellular retrograde pathways. This complexity makes retrograde transport an attractive therapeutic option, since the precise inhibition of one component of a particular pathway can result in a very specific impact on a process, such as virion wrapping, but leave other retrograde pathways functional and therefore minimize indirect effects on the cell.

Our work shows that GARP is required for at least one of the retrograde transport pathways used by VACV but does not rule out the possibility that VACV uses multiple retrograde transport pathways, some of which are independent of GARP.

The highly effective inhibition of VACV multistep replication and EEV production by the retrograde transport inhibitors Retro-1, Retro-2, and related compounds highlighted the importance of retrograde transport to poxviruses. These drugs have been shown to inhibit processes dependent on retrograde transport, such as the activity of ricin, Shiga toxin, and Shiga-like toxins 1 and 2 (26, 34), and the entry of AAV (22). The wrapping process of VACV is targeted by the two well-characterized antiorthopoxvirus drugs IMCBH [*N*(1)-isonicotinoyl-*N*(2)-3-methyl-4-chlorobenzoylhydrazine] and ST-246 {4-trifluoromethyl-*N*-[3,3a,4,4a,5,5a,6,6a-octahydro-1,3-di oxo-4,6-ethenocycloprop [f]isoindol-2(1H)-yl]-benzamide}. IMCBH is a selective inhibitor of VACV multiplication (45). It targets the viral protein F13, which is essential for wrapping but has no activity *in vivo* and is therefore not a candidate for therapeutic use (46). The more recently characterized drug, ST-246, also targets F13 (47, 48). Retro-1 and -2 target the same process (wrapping) as these two drugs *in vitro* and show the same virus replication profiles as ST-246-treated cells, namely, a 5- to 10-fold reduction in EEV production in a one-step growth curve, which equates to an almost complete reduction in multistep growth. However, Retro-1 and -2 exert their effect via an as yet unknown host protein rather than a viral protein and are therefore less susceptible to viral escape via mutations in the F13 gene, as seen in VACV strains which develop resistance to IMCBH and ST-246 (46, 48).

Retro-1 and Retro-2 have been shown to have *in vivo* efficacy against ricin (34), Shiga toxin produced by enterohemorrhagic Escherichia coli O104:H4 (36), and Leishmania (35) in mice. Since the drugs showed strong inhibition of VACV replication *in vitro*, we tested the efficacy of Retro-2 *in vivo* using an intranasal murine model of poxvirus disease. We compared two treatment regimes of 100 mg/kg given twice, either once before and once after infection (days −1 and +1) or twice after infection (days +1 and +2). Mice treated with Retro-2 prior to infection lost less weight and displayed less severe disease, and all mice survived the challenge. Pretreatment with Retro-2 therefore provided protection against poxviral disease, indicating that this family of small molecules may be developed as novel antipoxviral drugs for diseases such as cowpox and monkeypox and to supplement vaccination in response to a poxviral bioterrorism event.

### Note.

While the manuscript for the present study was under review, a study with broadly similar findings was published which described the reliance of VACV on cellular retrograde pathways for the production of IEVs (49).
